# Mindfulness training for reducing anger, anxiety, and depression in fibromyalgia patients

**DOI:** 10.3389/fpsyg.2014.01572

**Published:** 2015-01-12

**Authors:** Alberto Amutio, Clemente Franco, María de Carmen Pérez-Fuentes, José J. Gázquez, Isabel Mercader

**Affiliations:** ^1^Department of Social Psychology and Methodology of the Behavioral Sciences, Faculty of Psychology, University of the Basque CountrySan Sebastián, Spain; ^2^Department of Psychology, Faculty of Educational Sciences and Health, University of AlmeríaAlmeríaw, Spain

**Keywords:** fibromyalgia, anger, anxiety, depression, mindfulness

## Abstract

Fibromyalgia is a disabling syndrome. Results obtained with different therapies are very limited to date. The goal of this study was to verify whether the application of a mindfulness-based training program was effective in modifying anger, anxiety, and depression levels in a group of women diagnosed with fibromyalgia. This study is an experimental trial that employed a waiting list control group. Measures were taken at three different times: pretest, posttest, and follow-up. The statistical analyses revealed a significant reduction of anger (trait) levels, internal expression of anger, state anxiety, and depression in the experimental group as compared to the control group, as well as a significant increase in internal control of anger. It can be concluded that the mindfulness-based treatment was effective after 7 weeks. These results were maintained 3 months after the end of the intervention.

## INTRODUCTION

Fibromyalgia is an incapacitating syndrome of unknown etiology. Its main characteristic is chronic diffuse pain, mostly muscular. Other related symptoms are sensations of rigidity in the limbs, sleep problems, and fatigue (Rodero et al., 2009; Glattacker et al., 2010). Additionally, about 32.3% of fibromyalgia patients have an anxiety disorder, and 34.8% have a mood disorder, especially of a depressive type (Comeche et al., 2012; Parra et al., 2012). Likewise, anger toward oneself is high in these patients (Sayar et al., 2004). In addition, these negative emotions can increase or amplify pain (Sayar et al., 2004; van Middendorp et al., 2013). Thus, the way that people with fibromyalgia process and regulate their emotions may be relevant for adjustment to pain (Geenen et al., 2012).

The presence of anxiety and depressive symptoms is related to inadequate coping with stress. Fibromyalgia patients show a high amount of maladaptive coping strategies and a reduction of positive emotions (van Ittersum et al., 2009; Aparicio et al., 2011; van Middendorp et al., 2013), as well as a tendency to internalize and suppress anger. Moreover, some psychological variables related to the inability to cope, such as catastrophic thoughts, external locus of control, a low sense of self-efficacy, and feelings of helplessness are also present (Torres et al., 2009; Glattacker et al., 2010; Miró et al., 2012).

Different psychotherapeutic approaches have been used (medication, physical therapy, educational programs, behavior therapy, and cognitive-behavior therapy). Behavior therapy includes techniques such as thought-suppression, and cognitive-behavioral therapies use cognitive restructuring. Some research has found that, among patients scoring high on affect intensity, emotion expression was associated with less fibromyalgia impact (Geenen et al., 2012). However, other authors (Sayar et al., 2004) report that chronic angry emotional reactions are often maladaptive because they lead to pervasive interpersonal disruption and chronic sympathetic activation. Overall, the results obtained for reducing discomfort and other symptoms related to fibromyalgia are limited in the long-term (Rodero et al., 2009; Fjorback et al., 2012).

Difficulty in identifying and understanding feelings and emotions seems to be associated with fibromyalgia (Sayar et al., 2004). Consequently, awareness and acceptance of the sensations and feelings associated with chronic pain while remaining active may be another approach to the treatment of the symptomatology. Currently, one of the most promising techniques is mindfulness. Mindfulness can be described as non-judgmental attention to experiences in the present moment (Kabat-Zinn, 1990). It is both a practice and a way of being in the world (Carlson, 2012). Bishop et al. (2004) proposed a two-component model of mindfulness: self-regulation of attention and acceptance of one’s own experiences in a non-evaluative way (non-reactive awareness). In this sense, mindfulness is a valuable tool for learning new non-reactive models of responding to the emotional discomfort associated with different disorders, including fibromyalgia and to relate to pain without suffering.

Different researchers have shown the applicability of mindfulness techniques in education and health. Systematic reviews and meta-analyses (Chiesa and Serreti, 2009; Irwing et al., 2009; Carlson, 2012) reveal the effectiveness of these techniques, especially of mindfulness based stress reduction (MBSR; Kabat-Zinn, 1990) and mindfulness-based cognitive therapy (MBCT; Segal et al., 2002) in the reduction of stress-related symptoms (anxiety and depression, among others).

Given the decline in the quality of life that fibromyalgia patients are likely to face, it is especially important to undertake research studies proving the effectiveness of such programs. Hence, the main goal of this study was to verify the efficacy of a mindfulness program to reduce anger, anxiety, and depression levels in patients diagnosed with fibromyalgia.

## MATERIALS AND METHODS

### PARTICIPANTS

The final sample was comprised of 32 women with a diagnosis of fibromyalgia. They belonged to the Fibromyalgia Association of Almeria (AFIAL). Ages ranged from 39 to 66 years old (*M* = 51.82, SD = 10.18). Six percent of the sample did not have any studies, 63% had only primary studies, and 10% had completed higher studies. As for marital status, 82% were married, 3% divorced, and 3% were widows. From this pool of participants, 14 women were part of the experimental group and the remaining 18 were included in the control group.

### INSTRUMENTS

– *State-Trait Anger Expression Inventory* (*STAXI-2*; Spielberger, 1999; Spanish version by Miguel-Tobal et al., 2001). The STAXI-2 is a 49-item questionnaire containing the following scales: State Anger scale (It has three subscales: Angry Feelings, Physical Expression of Anger, Verbal Expression of Anger). It evaluates how often a person feels like Expressing Anger, whether verbally or physically; the Trait Anger scale (It has two subscales: Angry Temperament and Angry Reaction). It measures how often Angry Feelings are experienced over time; the Anger Expression and Anger Control scales assess four relatively independent anger-related traits (Anger Expression-Out, Anger Expression-In, Anger Control-Out, and Anger Control-In); and the Anger Expression Index: an overall measure of expression/control based on The Anger Expression and Anger Control Scales. As for reliability, alpha Cronbach values range from 0.89 to 0.71 for the Trait and State scales, respectively.

– *Beck Depression Inventory* (Beck et al., 1979; Spanish version by Vázquez and Sanz, 1999). The inventory is comprised of 21 items rated in a Likert-type scale ranging from 1 to 4, and with higher scores indicate higher levels of depression, and vice versa. It has been shown to have good internal consistency (α = 0.83).

– *State-Trait Anxiety Questionnaire (STAI*; Spielberger et al., 1983). The Spanish version of this questionnaire is made up of two subscales, each of them evaluating an independent aspect of anxiety: Trait Anxiety and State Anxiety. It has 40 items (20 for each scale), on which respondents have to evaluate themselves using a Likert-type scale ranging from 0 to 3. Individuals report how they usually feel (trait anxiety) and how they feel at the present moment (state anxiety). Internal consistency Cronbach alphas of 0.91 and 0.94 were obtained for Trait Anxiety and State Anxiety, respectively. Test–retest reliability of 0.81 and 0.40 were obtained for Trait Anxiety and State Anxiety, respectively (Echeburúa, 1993).

### DESIGN

The effectiveness of a mindfulness intervention program (independent variable) on anger, anxiety, and depression measures in fibromyalgia patients was analyzed by means of a quasi-experimental design (de la Torre et al., 2013; Morales et al., 2013), with measures at pretest/posttest/follow-up, using an experimental group and a waiting-list control group.

### PROCEDURE

First, we contacted the AFIAL association to offer a course entitled: “Training and practice of mindfulness in fibromyalgia patients.” Thirty-nine persons enrolled in the course. All participants completed the above-mentioned questionnaires individually, providing pretest measures of anger, anxiety, and depression levels. Next, participants were randomly assigned to one of the groups (experimental group, *n* = 20; control group, *N* = 19). Participants in the control group were informed that, due to space constraints, they would receive the course at a later time. Seven participants were excluded from the study (six subjects from the experimental group and one subject from the control group) either because they did not complete the course, or filled out the required questionnaires.

The mindfulness intervention program was applied to the experimental group (Franco, 2009), with one weekly 2-h session for a period of seven consecutive weeks. The training program was partially based on some of the elements and exercises of Kabat-Zinn (1990), mindfulness-related strategies used in acceptance and commitment therapy (Hayes et al., 1999; Wilson and Luciano, 2002; Carrascoso, 2006) along with explanations and discussions of the metaphors and exercises used in this therapy, and stories and tales taken from Zen philosophy (Deshimaru, 2006), and from Vipassana meditation (Hart, 1988).

The main goal of this intervention program is for patients to learn to let their thoughts flow, without trying to change them. Thus, learning not to think is not the aim, but instead the re-education of conditioned and automatic ways of reacting when faced with normally occurring internal and external events. Therefore, instead of trying to fight thoughts, emotions, and sensations, patients are encouraged to let them be and observe how they come and go. In this way, they learn how to break the habit of letting their thoughts, emotions, and sensations control them. Patients become aware of their presence, but they do not dwell on them, either on their content or veracity, considering them as events that are constantly flowing.

The program was taught by an experienced instructor in mindfulness. Training sessions used the following structure:

– Participants’ reflections about their mindfulness meditation exercise practice during the week (e.g., experiences, questions, obstacles).

– Practice of body-scan for 10 min.

– Presentation of several metaphors through different animations and stories and also some exercises for each of the sessions [i.e., *Unwelcome Party Guest metaphor*, *It’s a Beautiful Day metaphor*; Observing physical sensations of different body parts, breathing exercises, observing thoughts, accepting uncomfortable private events – See Hayes et al. (1999) and Wilson and Luciano (2002)].

– Practice of mindfulness, attending to the breath, for 30 min.

In order to facilitate the implementation of the program, patients were requested to practice the body-scan exercise daily for 10 min and the mindfulness breathing exercises (abdominal breathing) for 30 min. In addition, each participant had to record the practice of the assigned exercises using a register sheet especially designed for this purpose. Regarding compliance, 71% of the participants reported having practiced the body-scan, and 83% practiced the mindfulness breathing exercise.

Once the intervention was completed, posttest measures were taken in both groups to verify possible significant changes in anxiety, depression, and anger levels. Likewise, and with the aim of verifying whether the changes achieved were maintained over time, a follow-up evaluation was performed 3 months later, following the same procedure as in previous pretest and posttest phases. After completing this follow-up evaluation, the mindfulness course was taught to the control group, as they had been informed at the beginning of this research project.

All participants were informed of the goal of the study and written informed consent was obtained from them to use the data obtained. Confidentiality and anonymity were guaranteed. The Committee of Bioethics of the University of Almería approved the investigation.

### STATISTICAL ANALYSES

The data obtained were not normally distributed and were non-parametric. The Mann–Whitney *U*-test for independent samples was used to determine the presence of statistically significant group differences in each of the study phases and in all the components and dimensions of the evaluated variables (González-Cabanach et al., 2013). Bonferroni corrections were also applied in order to reduce the probability of finding statistical differences due to chance.

Due to the non-normal distribution of the data, Friedman test for related samples was used to determine possible statistically significant differences in the control and experimental groups in the means of the variables among the different phases of the study. Additionally, Wilcoxon’s non-parametric test for dependent samples was used to determine if the obtained differences were between pretest and posttest scores, pretest/follow-up, and/or posttest/follow-up.

Finally, to assess the magnitude of change exhibited by the experimental group after the intervention according to the posttest and maintenance phase measures, Cohen’s *d* (Cohen, 1988) was also calculated as an indicator of the effect of the intervention. Data was analyzed with the SPSS, version 22.0.

## RESULTS

First, means and SDs for all the variables were calculated in both groups (see **Tables 1** and **2**.

**Table 1:** Mean and SD of the target variables at pretest, posttest, and follow-up in the experimental group.

	Pretest		Posttest		Follow-up	
Variables	*M*	SD	*M*	SD	*M*	SD
State Anger	19.09	6.48	16.50	5.44	17.53	5.13
* Angry Feelings*	7.86	3.97	6.03	3.44	6.57	3.27
* Physical Expression of Anger*	5.21	2.42	5.14	1.53	5.43	1.91
* Verbal Expression of Anger*	5.93	3.47	5.36	2.81	5.53	2.98
Trait Anger	25.02	5.34	23.02	5.83	23.61	5.63
* Angry Temperament*	11.57	2.37	10.57	2.43	10.91	2.29
* Angry Reaction*	13.43	3.67	12.45	3.91	12.70	3.80
Anger Expression-Out	8.43	2.06	7.79	2.04	8.14	4.47
Anger Expression-In	13.71	4.33	10.02	3.81	10.86	5.95
Anger Control-Out	18.07	4.73	18.43	4.68	19.07	7.41
Anger Control-In	18.86	5.33	21.36	5.54	20.79	5.26
Anger Expression Index	21.21	8.85	22.57	8.42	21.86	8.59
State Anxiety	38.63	8.75	29.29	9.69	27.85	8.14
Trait Anxiety	35.81	9.61	32.29	8.53	31.71	7.93
Depression	41.79	8.96	36.02	7.49	35.12	8.26

**Table 2:** Mean and SD of the target variables at pretest, posttest, and follow-up in the control group.

	Pretest		Posttest		Follow-up	
Variables	*M*	SD	*M*	SD	*M*	SD
State Anger	20.14	9.78	20.43	9.46	21.78	8.01
* Angry Feelings*	8.07	3.54	8.21	3.64	8.57	3.48
* Physical Expression of Anger*	6.50	3.52	6.93	3.17	7.07	3.10
* Verbal Expression of Anger*	5.57	4.11	5.29	4.08	6.14	2.95
Trait Anger	24.50	7.03	24.81	6.41	25.72	6.14
* Angry Temperament*	11.43	4.10	11.96	3.86	12.23	4.05
* Angry Reaction*	13.07	4.74	12.85	4.43	13.29	3.91
Anger Expression-Out	9.07	2.70	10.14	2.77	10.01	3.06
Anger Expression-In	13.79	3.68	14.29	3.75	14.71	3.68
Anger Control-Out	16.57	4.63	16.29	5.38	16.36	5.70
Anger Control-In	15.64	3.56	15.14	3.95	14.86	4.23
Anger Expression Index	27.14	11.45	28.71	9.06	29.15	9.96
State Anxiety	39.93	8.34	41.12	9.25	40.29	7.89
Trait Anxiety	34.03	7.58	36.24	8.98	34.97	9.37
Depression	40.15	9.19	41.87	10.36	42.68	9.79

Man–Whitney’s *U* test for independent samples of pretest scores revealed that there were no statistically significant group differences in the mean scores of the target variables. However, significant differences between the means of posttest scores were revealed for many variables. Specifically, significant lower scores were observed in the experimental group on State Anger (*Z* = -3.57; *p* = 0.001), Angry Feelings (*Z* = -3.16; *p* = 0.005), Anger Expression-In (*Z* = -4.32; *p* = 0.000), State-Anxiety (*Z* = -5.43; *p* = 0.000), and Depression (*Z* = -3.26; *p* = 0.005), and higher scores in internal control of anger (*Z* = -3.41; *p* = 0.001) when compared to the control group. Likewise, mean group comparisons performed at the follow-up phase revealed similar significant differences when compared to the posttest phase, except for Angry Feelings. Bonferroni correction was also used in order to reduce the probability of finding statistical differences due to chance. A significance level of 0.0083 was applied (**Table 3**).

**Table 3:** Group differences of target variables at pretest, posttest, and follow-up (Mann–Whitney *U* Test for independent samples).

	Pretest		Posttest		Follow-up	
Variables	*z*	*p*	*z*	*p*	*z*	*p*
State Anger	1.27	0.221	-3.57	0.001**	-3.79	0.001**
* Angry Feelings*	0.85	0.480	-3.16	0.005*	-2.97	0.018
* Physical Expression of Anger*	1.35	0.218	1.57	0.161	1.77	0.056
* Verbal Expression of Anger*	1.14	0.265	1.45	0.181	1.07	0.281
Trait Anger	1.24	0.226	1.11	0.156	1.17	0.259
* Angry Temperament*	1.46	0.191	1.66	0.062	1.71	0.058
* Angry Reaction*	1.54	0.175	1.58	0.071	1.63	0.065
Anger Expression-Out	0.77	0.512	1.16	0.261	1.21	0.148
Anger Expression-In	0.54	0.898	-4.32	0.000***	-3.91	0.000***
Anger Control-Out	0.84	0.486	1.12	0.269	1.36	0.120
Anger Control-In	1.26	0.234	-3.41	0.001**	-3.24	0.005*
Anger Expression Index	0.83	0.488	1.23	0.228	0.97	0.412
State Anxiety	0.73	0.532	-5.43	0.000***	-5.78	0.000***
Trait Anxiety	0.69	0.587	-2.45	0.065	-2.97	0.054
Depression	0.57	0.908	-3.26	0.005*	-3.47	0.001**

*p = ≤ 0.0083; **p = 0.001; ***p < 0.0001.

Next, Friedman test for related samples was also performed in the experimental group. In order to reduce the probability of finding statistical differences due to chance Bonferroni correction was applied, considering a significance level equal or lower to 0.017. Significant differences were found in State Anger (χ^2^ = 15.18, *p* = 0.001), internal expression of anger (χ^2^ = 16.12, *p* = 0.000), internal control of Anger (χ^2^ = 8.29, *p* = 0.016), Trait Anxiety (χ^2^ = 25.08, *p* = 0.000), and Depression (χ^2^ = 14.78, *p* = 0.001; **Table 4**).

**Table 4:** Friedman test for related simples in the experimental group in the studied variables.

Variables	χ^2^	*p*
State Anger	15.18	0.001**
* Angry Feelings*	7.08	0.029
* Physical Expression of Anger*	3.25	0.197
* Verbal Expression of Anger*	4.41	0.110
Trait Anger	3.79	0.149
* Angry Temperament*	4.46	0.112
* Angry Reaction*	3.71	0.156
Anger Expression-Out	5.16	0.76
Anger Expression-In	16.12	0.000***
Anger Control-Out	5.31	0.071
Anger Control-In	8.29	0.016*
Anger Expression Index	4.14	0.0129
State Anxiety	25.08	0.000***
Trait Anxiety	7.91	0.022
Depression	14.78	0.001**

*p = 0.016; **p ≤ 0.017; ***p < 0.001.

In addition, Wilcoxon’s test for related samples comparing the pretest and posttest mean scores of the experimental group revealed significant reductions in several dimensions of anger: State Anger (*Z* = -4.92; *p* = 0.000), internal expression of anger (*Z* = -4.43; *p* = 0.000), and internal control of anger (*Z* = 2.87; *p* = 0.013), as well as in State Anxiety (*Z* = -6.18; *p* = 0.000), and Depression (*Z* = -3.79; *p* = 0.001), whereas a significant increase in internal control of anger was found (*Z* = 2.87, *p* = 0.0013). Similarly, when comparing mean pretest scores with mean follow-up scores, significant differences were also obtained in the same variables and dimensions. However, no significant differences were found between mean posttest and follow-up scores for any of the target variables of the experimental group (**Table 5**).

**Table 5:** Pretest–posttest, pretest–follow-up, and posttest–follow-up differences in the experimental group (Wilcoxon test for related samples).

	Pretest–posttest		Pretest–follow-up		Posttest–follow-up	
Variables	*z*	*p*	*z*	*p*	*z*	*p*
State Anger	-4.92	0.000****	-3.03	0.011**	0.87	0.396
Anger Expression-In	-4.43	0.000****	-3.37	0.005**	1.08	0.325
Anger Control-In	2.87	0.013**	2.73	0.017*	1.47	0.189
State Anxiety	-6.18	0.000****	-6.79	0.000****	0.93	0.306
Depression	-3.79	0.001***	-3.93	0.001***	0.78	0.491

*p = 0.017; **p < 0.017; ***p = 0.001; ****p < 0.0001.

Conversely, Friedman test for related samples in the control group revealed no significant differences (see **Table 6**).

**Table 6:** Friedman test for related simples in the control group in the studied variables.

Variablesχ2	*p*
State Anger	2.94	0.230
* Angry Feelings*	2.26	0.323
* Physical Expression of Anger*	4.31	0.117
* Verbal Expression of Anger*	1.03	0.597
Trait Anger	3.39	0.214
* Angry Temperament*	4.01	0.142
* Angry Reaction*	1.11	0.572
Anger Expression-Out	4.13	0.132
Anger Expression-In	4.33	0.115
Anger Control-Out	0.78	0.677
Anger Control-In	4.97	0.083
Anger Expression Index	3.45	0.211
State Anxiety	2.45	0.307
Trait Anxiety	1.87	0.396
Depression	3.04	0.225

Cohen’s *d* (Cohen, 1988) was used to evaluate the effect size at posttest, as well as at the follow-up assessment. Values above 1.5 indicate the presence of very important changes, values between 1.5 and 0.5 indicate moderate changes, values between 0.5 and 0.2 indicate a low change, and values below 0.2 indicate a very low change. In **Table 7**, Cohen’s *d* scores for posttest measures show important changes in internal Expression of Anger and State Anxiety; moderate changes in State Anger, Angry Feelings, Angry Temperament, internal control of anger, and Depression; low changes in Verbal Expression of Anger, Trait Anger, Anger Reaction, External Expression of Anger, Anger Expression Index, and Trait Anxiety; and very low changes in physical expression and external control of anger.

**Table 7:** Cohen’s *d* and pretest–posttest and pretest–follow-up percentages of change in the experimental group.

	Cohen’s *d*		% of Change	
Variables	Posttest	Follow-up	Pretest–posttest	Posttest–follow-up
State Anger	0.61	0.37	-13.56	-8.17
* Angry Feelings*	0.69	0.50	-23.28	-16.41
* Physical Expression of Anger*	0.04	0.14	-1.34	4.22
* Verbal Expression of Anger*	0.25	0.17	-9.61	-6.74
Trait Anger	0.25	0.36	-3.99	-5.36
* Angry Temperament*	0.59	0.41	-8.64	-5.71
* Angry Reaction*	0.36	0.27	-7.29	-5.43
Expression-Out	0.44	0.11	-7.59	-3.44
Expression-In	1.28	0.77	-26.91	-20.78
Control-Out	0.11	0.23	1.99	5.53
Control-In	0.63	0.51	13.25	10.23
Anger Expression Index	0.22	0.10	6.41	3.06
State Anxiety	1.01	1.27	-24.18	-27.91
Trait Anxiety	0.39	0.47	-9.83	-11.45
Depression	0.68	0.77	-13.81	-15.96

Regarding the amount of change in the mean scores at pretest and follow-up, we observed important changes in State Anxiety; moderate changes in Angry Feelings, internal expression and internal control of anger, and Depression; low changes in state and trait anger, Angry Temperament, Angry Reaction, External Control of Anger, and Trait Anxiety; and very low changes in physical expression, verbal expression, external expression of anger, and in the anger expression index (see **Table 7**).

Finally, percentages of change between pretest and posttest measures, and between pretest and follow-up were calculated. As shown in **Table 7**, change between pretest and posttest measures showed an increase of 13% in internal anger control, as well as reductions ranging between 13 and 27% in State Anger, including Angry Feelings, Depression, State Anxiety, and internal expression of anger. The percentage of change between pretest and follow-up measures showed a 10% increment in internal control of anger, and reductions ranging between 28 and 16% in State-Anxiety, Depression, Angry Feelings, and internal expression of anger.

## DISCUSSION

Findings confirm that the mindfulness-based training program was effective. After 7 weeks, patients achieved significantly lower scores in the following variables: State Anger, internal anger expression, State Anxiety, and Depression. Significant improvements were also attained in the internal control of anger. In contrast, the control group did not obtain any significant result in these variables. Moreover, the experimental group maintained many of the benefits throughout the follow-up phase, especially for depression, state anxiety, and internal expression and control of anger. The improvement obtained in the internal control of anger had a medium effect size. Remarkably, important improvements were obtained for state anxiety, which increased after the 3-months follow-up period.

These findings are relevant if we take into account that, to date, treatments for this disease, especially pharmacological treatments, have proven unsatisfactory due to lack of adherence and to relapses (Fjorback et al., 2012). The improvements obtained in internal control and expression of anger are especially important when considering that fibromyalgia patients use maladaptive strategies, such as internalizing and suppressing anger. In contrast, research has found that emotional expression and control through adaptive coping strategies is associated with lower impact of fibromyalgia (Geenen et al., 2002; Sayar et al., 2004; van Ittersum et al., 2009). Also, the changes in anxiety and depression are consistent with previous investigations associating the practice of mindfulness with important reductions in these variables and an increase in the experience of positive emotions (Segal et al., 2002; Grossman et al., 2007; Amutio et al., 2013; Parra and Latorre, 2013).

By practicing mindfulness, individuals can learn to maintain a relaxed mind and concentrate on the present moment. An integral part of the practice is to cultivate an attitude of kindness, acceptance, generosity, and patience toward unpleasant emotions and thoughts that may arise. Concretely, and for depression, mindfulness practice helps patients to interrupt depressogenic reactions toward pain and other related symptoms. Patients learn to accept their pain-related automatic thoughts (including catastrophizing) with a non-judgmental attitude of openness, minimizing behavioral avoidance (Segal et al., 2002). Some studies have shown that the development of acceptance and self-compassion are important mediating variables in the positive effects of mindfulness on anxiety and depression (Kuyken et al., 2010; Schütze et al., 2010; Luciano et al., 2014). At the same time, the experience of positive emotional states associated with mindfulness can provoke a modification of helplessness-related beliefs (Amutio and Smith, 2008) and a higher motivation to continue with the treatment.

Unlike other therapies, mindfulness-based interventions provide techniques, abilities, and perspectives that patients are encouraged to practice and that, once learned, can be applied to everyday life. Furthermore, mindfulness training is not aimed at symptom reduction but more basically toward altering the way that mental processes and content are experienced. Development of acceptance may enhance well-being, even in the face of continued symptoms.

Nevertheless, our study presents the limitation of not having an alternative treatment for the control group. Taking into account the heterogeneity of this disorder and the variability among patients, studies with active control groups are clearly needed to compare the efficacy of mindfulness-based interventions with different treatments, such as cognitive-behavior therapy and physical training programs and to determine the typologies of patients who could benefit from a certain treatment.

The findings reveal the feasibility of mindfulness-based treatment intervention on anger, a variable on which few studies have been carried out to date. They also add to the growing body of literature documenting the effectiveness of mindfulness for the treatment of anxiety and depression. Finally, one of the main advantages of this program is that it can be taught in a group format, which reduces the costs for participants. It also optimizes the economic and social resources invested in health.

## Conflict of Interest Statement

The authors declare that the research was conducted in the absence of any commercial or financial relationships that could be construed as a potential conflict of interest.

## References

[B1] Amutio A. Martínez-Taboada C. Mozaz M. J. (2013). “Effective clinical use of mindfulness for increasing physicians, well-being levels: a one-year study,” in *Proceeding of the First International Conference on Mindfulness (ICM)* Rome.

[B2] Amutio A. Smith J. C. (2008). Stress and irrational beliefs in college students. *Ansiedad Estrés* 14 211–220.

[B3] Aparicio M. Ramos-Cejudo J. Salguero J. Sanz-Blasco R. (2011). Trait anxiety and self-rated health as predictor variables of medical attention in Spanish population. *Ansiedad Estrés* 17 125–136.

[B4] Beck A. T. Rush A. J. Shaw B. F. Emery G. (1979). *Cognitive Therapy of Depression.* New York: Guilford Press.

[B5] Bishop S. R. Lau M. Shapiro S. Carlson L. E. Anderson N. Carmody J. (2004). Mindfulness: a proposed operational definition. *Clin. Psychol.* 11 230–241 10.1093/clipsy.bph077

[B6] Carlson L. E. (2012). Mindfulness-based interventions for physical conditions: a narrative review evaluating levels of evidence. *ISRN Psychiatry* 2012:651583 10.5402/2012/651583 PMC3671698 23762768

[B7] Carrascoso F. J. (2006). *Terapia de Aceptación y Compromiso: Características, Técnicas Clínicas Básicas y Hallazgos Empíricos* [Therapy of Acceptance and Commitment: Characteristics, Basic Clinical Techniques and Empirical Findings]. *Behav. Psychol.* 14 361–386.

[B8] Chiesa A. Serreti A. (2009). Mindfulness-based stress reduction for stress management in healthy people: a review and meta-analysis. *J. Altern. Complement. Med.* 15 593–600 10.1089/acm.2008.0495 19432513

[B9] Cohen J. (1988). *Statistical Power Analysis for the Behavioral Sciences* 2nd Edn Hillsdale, NJ: Erlbaum.

[B10] Comeche M. I. Ortega J. Rodríguez M. F. Díaz M. I. Vallejo M. A. (2012). Structure and adequacy of the Beck Depression Inventory in patients with fibromyalgia. *Psicothema* 24 668–673. 23079368

[B11] de la Torre M. J. Casanova P. F. Carpio M. V. Cerezo M. T. (2013). *Consistencia e Inconsistencia Parental: Relaciones con la Conducta Agresiva y Satisfacción Vital de los Adolescentes* [Perceived Interparental-Consistency: Relationships Between Aggressive Behavior and Life Satisfaction in Adolescents]. *Eur. J. Educ. Psychol.* 6 135–149 10.1989/ejep.v6i2.112

[B12] Deshimaru T. (2006). *La Práctica del Zen* [The Practice of Zen]. Barcelona: RBA.

[B13] Echeburúa E. (1993). *Ansiedad Crónica. Evaluación y Tratamiento* [Chronic Anxiety. Assessment and Treatment]. Salamanca: Eudema.

[B14] Fjorback L. O. Arendt M. Ornbol E. Walach H. Rehfeld E. Schröder A. (2012). Mindfulness therapy for somatization disorder and functional somatic syndromes: randomized trial with one-year follow-up. *J. Psychosom. Res.* 74 31–40 10.1016/j.jpsychores.2012.09.006 23272986

[B15] Franco C. (2009). *Meditación Fluir para Serenar el Cuerpo y la Mente* [Flowing Meditation to Calm Body and Mind]. Madrid: Bubok.

[B16] Geenen R. Jacobs J. W. Bijlsma J. W. (2002). Evaluation and management of endocrine dysfunction in fibromyalgia. *Rheum. Dis. Clin. North Am.* 28 389–404 10.1016/S0889-857X(01)00009-6 12122926

[B17] Geenen R. Van Ooijen-van der Linden L. Lumley M. A. Bijlsma J. W. J. Van Middendorp H. (2012). The match-mismatch model of emotion processing styles and emotion regulation strategies in fibromyalgia. *J. Psychosom. Res.* 72 45–50 10.1016/j.jpsychores.2011.09.004 22200522

[B18] Glattacker M. Opitz U. Jäckel W. H. (2010). Illness representations in women with fibromyalgia. *Br. J. Health Psychol.* 15 367–387 10.1348/135910709X466315 19646332

[B19] González-Cabanach R. Fari na F. Freire C. González P. Ferradás M. M. (2013). *Diferencias en el Afrontamiento del Estrés en Estudiantes Universitarios Hombres y Mujeres* [Differences in Coping Between Men and Women University Students]. *Eur. J. Educ. Psychol.* 6 19–32 10.1989/ejep.v6i1.100

[B20] Grossman P. Tiefenthaler-Gilmer U. Raysz A. Kesper U. (2007). Mindfulness training as an intervention for fibromyalgia: evidence of post-intervention and 3-year follow-up benefits in well-being. *Psychother. Psychosom.* 76 226–233 10.1159/000101501 17570961

[B21] Hart W. (1988). *The Art of Living: Vipassana Meditation. Mumbai: Embassy Books* [Spanish translation: *La Vipasana. El arte de la Meditación*. Madrid: Luz de Oriente, 1994].

[B22] Hayes S. C. Stroshal K. D. Wilson K. G. (1999). *Acceptance and Commitment Therapy.* New York: Guilford Press.

[B23] Irwing J. A. Dobkin P. L. Park J. (2009). Cultivating mindfulness in health care professionals: a review of empirical studies of mindfulness-based stress reduction (MBSR). *Complement. Ther. Clin. Pract.* 15 61–66 10.1016/j.ctcp.2009.01.002 19341981

[B24] Kabat-Zinn J. (1990). *Full Catastrophe Living: Using the Wisdom of your Body and Mind to Face Stress, Pain and Illness*. New York: Delacorte.

[B25] Kuyken W. Watkins E. Holden E. White K. Taylor R. S. Byford S. (2010). How does mindfulness-based cognitive therapy work? *Behav. Res. Ther.* 48 1105–1112 10.1016/j.brat.2010.08.003 20810101

[B26] Luciano J. V. Guallar J. A. Aguado J. López-del-Hoyo Y. Olivan B. Magallón R. (2014). Effectiveness of group acceptance and commitment therapy for fibromyalgia: a 6-month randomized controlled trial (EFFIGACT study). *Pain* 155 693–702 10.1016/j.pain.2013.12.029 24378880

[B27] Miró E. Diener F. N. Martínez M. P. Sánchez A. I. Valenza M. C. (2012). Fibromyalgia in men and women: comparison of the main clinical symptoms. *Psicothema* 24 10–15. 22269357

[B28] Morales F. M. Trianes M. V. Casado A. M. (2013). Effectiveness of a programme to promote the acquisition of solidarity in university students. *Eur. J. Educ. Psychol.* 6 95–104 10.1989/ejep.v6i2.106

[B29] Parra M. Latorre J. M. (2013). Effectiveness of mindfulness-based cognitive therapy in the treatment of fibromyalgia: a randomised trial. *Cogn. Ther. Res.* 37 1015–1026 10.1007/s10608-013-9538-z

[B30] Parra M. Latorre J. M. Monta nés J. (2012). Mindfulness-based cognitive therapy and reduction of anxiety symptoms in people with fibromyalgia. *Ansiedad Estrés* 18 141–154 10.4103/0973-6131.113405

[B31] Rodero B. García-Campayo J. Casanueva B. Buriel Y. (2009). Non-pharmacological treatments of fibromyalgia: a current review. *Rev. Psicopatol. Psicol. Clín.* 14 137–151.

[B32] Sayar K. Gulee H. Topbas M. (2004). Alexithymia and anger in patients with fibromyalgia. *Clin. Rheumatol.* 23 441–448 10.1007/s10067-004-0918-3 15278756

[B33] Schütze R. Rees C. Preece M. Schütze M. (2010). Low mindfulness predicts pain catastrophizing in a fear-avoidance model of chronic pain. *Pain* 148 120–127 10.1016/j.pain.2009.10.030 19944534

[B34] Segal Z. V. Williams J. M. Teasdale J. D. (2002). *Mindfulness-Based Cognitive Therapy for Depression: A New Approach for Preventing Relapse.* New York: Guilford Press. [Spanish translation: *Terapia Cognitiva de la Depresión Basada en la Consciencia Plena: Un Nuevo Abordaje Para la Prevención de Recaídas*. Bilbao: Desclée, 2006].

[B35] Spielberger C. D. (1999). *State-Trait Anger Expression Inventory-2: STAXI-2 Professional Manual*. Odessa, FL: Psychological Assessment Resources [Spanish version: Miguel-Tobal, J., Casado, M., Cano, A., and Spielberg, C. (2001). *Inventario de Expresión de Ira Estado-Rasgo STAXI-2*. Madrid: TEA].

[B36] Spielberger C. D. Gorsuch R. L. Lushene R. E. (1983). *Manual for the State-Trait Anxiety Inventory.* Palo Alto, CA: Consulting Psychologists Press.

[B37] Torres X. Collado A. Arias A. Peri J. M. Bailles E. Salamero M. (2009). Pain locus of control predicts return to work among Spanish fibromyalgia patients after completion of a multidisciplinary pain program. *Ann. Gen. Hosp. Psychiatry* 31 137–145 10.1016/j.genhosppsych.2008.12.001 19269534

[B38] van Ittersum M. W. van Wilgen C. P. Hilberdink W. K. H. A. Groothoff J. W. van der Schans C. P. (2009). Illness perceptions in patients with fibromyalgia. *Patient Educ. Couns.* 74 53–60 10.1016/j.pec.2008.07.041 18815004

[B39] van Middendorp H. Lumley M. A. Houtveen J. H. Jacobs J. W. G. Bijlsma J. W. J. Geenen R. (2013). The impact of emotion-related autonomic nervous system responsiveness on pain sensitivity in female patients with fibromyalgia. *Psychosom. Med.* 75 765–773 10.1097/PSY.0b013e3182a03973 23922401

[B40] Vázquez C. Sanz J. (1999). Reliability and validity of the Spanish version of Beck’s Depression Inventory (1978) in patients with psychological disorders. *Clín. Salud.* 10 59–81.

[B41] Wilson K. G. Luciano M. C. (2002). *Terapia de Aceptación y Compromiso. Un Tratamiento Conductual Orientado a Los Valores* [Acceptance and Commitment Therapy: A Behavioral Therapy Oriented To Values]. Madrid: Pirámide.

